# Suppressing the Effect of the Wetting Layer through AlAs Capping in InAs/GaAs QD Structures for Solar Cells Applications

**DOI:** 10.3390/nano12081368

**Published:** 2022-04-15

**Authors:** Nazaret Ruiz, Daniel Fernández, Lazar Stanojević, Teresa Ben, Sara Flores, Verónica Braza, Alejandro Gallego Carro, Esperanza Luna, José María Ulloa, David González

**Affiliations:** 1 University Research Institute on Electron Microscopy & Materials, (IMEYMAT), Universidad de Cádiz, 11510 Puerto Real, Spain; teresa.ben@uca.es (T.B.); sara.flores@uca.es (S.F.); veronica.braza@uca.es (V.B.); david.gonzalez@uca.es (D.G.); 2 Institute for Systems Based on Optoelectronics and Microtechnology (ISOM), Universidad Politécnica de Madrid, Avda. Complutense 30, 28040 Madrid, Spain; lazar@isom.upm.es (L.S.); alejandro.gallego@alumnos.upm.es (A.G.C.); jmulloa@isom.upm.es (J.M.U.); 3 Paul-Drude-Institut für Festkörperelektronik, Leibniz-Institut im Forschungsverbund Berlin e.V., Hausvogteiplatz 5-7, D-10117 Berlin, Germany; luna@pdi-berlin.de

**Keywords:** InAs quantum dots solar cells, AlAs capping, (S)TEM

## Abstract

Recently, thin AlAs capping layers (CLs) on InAs quantum dot solar cells (QDSCs) have been shown to yield better photovoltaic efficiency compared to traditional QDSCs. Although it has been proposed that this improvement is due to the suppression of the capture of photogenerated carriers through the wetting layer (WL) states by a de-wetting process, the mechanisms that operate during this process are not clear. In this work, a structural analysis of the WL characteristics in the AlAs/InAs QD system with different CL-thickness has been made by scanning transmission electron microscopy techniques. First, an exponential decline of the amount of InAs in the WL with the CL thickness increase has been found, far from a complete elimination of the WL. Instead, this reduction is linked to a higher shield effect against QD decomposition. Second, there is no compositional separation between the WL and CL, but rather single layer with a variable content of InAlGaAs. Both effects, the high intermixing and WL reduction cause a drastic change in electronic levels, with the CL making up of 1–2 monolayers being the most effective configuration to reduce the radiative-recombination and minimize the potential barriers for carrier transport.

## 1. Introduction

The cost of electricity produced from a photovoltaic system depends directly on the efficiency of the solar cells (SC), so the main driver of innovation in this field is focused on increasing it. Although theory predicts for a single-junction solar cell a maximum efficiency of up to 85%, the Shockley–Queisser limit lowers this energy conversion efficiency to below 31% [1]. The two main physical processes involved are thermal dissipation losses and non-absorption of photons below the bandgap energy of the material of the cell. Therefore, the next generation of SC with high conversion efficiency needs to develop methods to reduce these losses. Among them, intermediate bandgap solar cells (IBSCs) have attracted a great deal of attention since the concept was first put forward in 1997 [2]. This concept is based on the absorption of energy photons below the bandgap by an “intermediate” electronic band (IB) suitably situated between the conduction and valence bands (CB and VB) that preserves the cell output voltage. Theoretical calculation of the conversion efficiency based on the ideal detailed balance model predicts 47% under one sun and 63% under the maximum concentration [3].

Among the different strategies to introduce intermediate levels, a promising one is to take advantage of nanostructures such as quantum dots (QDs) or quantum wells (QWs) [3,4,5]. In this framework, self-assembled (Al)GaAs/InAs/GaAs QDs arrays grown via epitaxial Stranski–Krastanov (SK) mode within the intrinsic region of a GaAs pin diode have been widely used as a means of implementing the concept of IBSCs due to their discrete density of states and well-established technology, where the IB arises from the energy states associated to the electrons confined in the CB of the QDs [6,7]. Although several of the principles of IBSC operation have been demonstrated [8,9], the reported efficiencies are nowadays well below expectations [10,11]. Several issues need to be overcome in QDSCs, such as to avoid an important reduction of the open-circuit voltage (*V*_OC_) that leads to a drop in the efficiency in comparison to their single junction GaAs SC counterparts [3,12]. It is said that the key prerequisite for improvement of the output voltage is the suppression of photoelectron capture from the conduction band into QD states which mainly occurs through the extended wetting layer (WL) state [13,14]. Certainly, it is demonstrated that the large phase volume of WL plays a major role in this *V*_OC_ reduction [15], so QD properties can be radically altered if WL states are non-existent. It has been proposed that conventional SK QDs should be advantageously replaced with their no-WL counterparts [16,17].

Recently, the use of thin AlAs capping layers (CLs) on InAs QDs has attracted special attention since important improvements [18,19] or even a complete recovery [14] of the *V*_OC_ in these QDSCs have been reported. It has been proposed that the cause of this improvement is the elimination of the In(Ga)As WL due to phase separation [20,21]. This hypothesis comes from earlier studies by Tsatsul’nikov et al. [22] using cross-sectional transmission electron microscopy (TEM) imaging. The dark contrast under g002 dark field (DF) conditions of the WL in InAs/GaAs QDs layers is replaced by a white contrast in case of AlAs/InAs/GaAs QDs [14,18]. They suggested that the interpretation of these images is that the In in the WL in the regions between the QDs is strongly depleted or almost completely gone. For them, the only possible explanation for this behavior is that there is a replacement of the In atoms in the WL by Al atoms of the CL, followed by a surface migration of In into the uncapped QDs like what occurs during the formation of QDs. This reasoning has been used by several authors to explain the performance improvements observed in QDSC with this system [14,23].

However, the mechanism proposed in this model is controversial. First, it is true that phenomena of atomic surface exchange could occur during epitaxial growth, where certain atoms in the subsurface are promoted to the surface being substituted by other newcomers [24,25,26,27]. Although the strong surface segregation of In that happens during the GaAs capping of InAs QDs is well-known [28,29], there is no consensus on the role that AlAs capping could play in this system. Indeed, some authors proposed that AlAs CL strongly suppresses both In segregation and In–Ga intermixing [30,31], while others pointed to a similar behavior compared to GaAs capping [32,33]. Second, the proposed surface migration of In toward the QDs that removes the WL, like what occurs during the QD formation, is opposite to the process observed during QD capping. In fact, it is broadly admitted that a massive transport of In atoms occur from the dots to the WL during capping due to a decomposition of QD [34,35].

The aim of this work is to shed light on the structural and compositional changes at the nanometer level that occur in the WL during AlAs capping of InAs QD for SC applications. For this purpose, analyses have been performed using different CL thicknesses of AlAs combining different state-of-the-art scanning (S)TEM techniques. As will be shown in the manuscript, capping with AlAs does not lead to a complete removal of the WL, but to a large intermixing between the CL and the WL that substantially alters the energetic states associated with it. These results further clarify the controversy over the interpretation of the DCTEM g002 DF images that existed.

## 2. Materials and Methods

**Figure 1:**
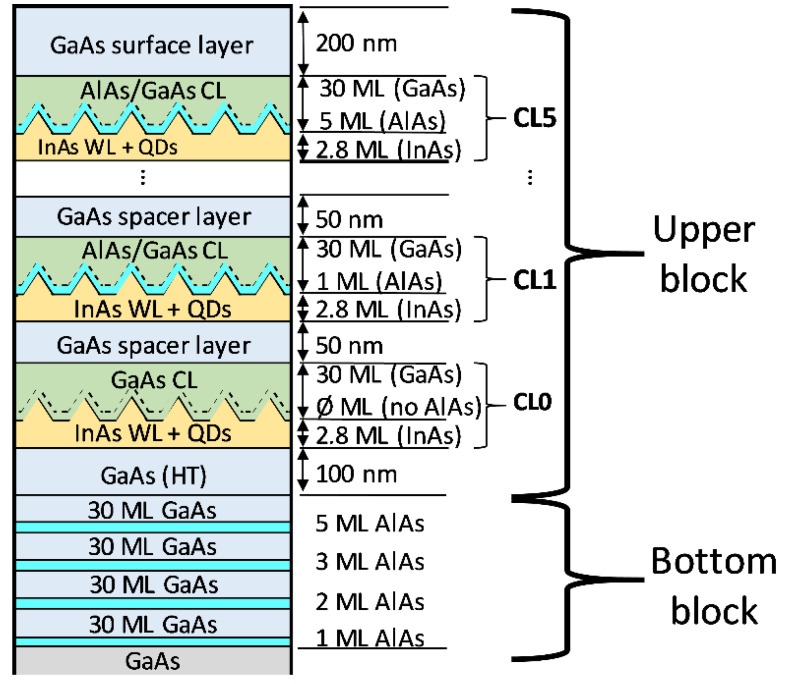
Scheme of the sample. In the first block, AlAs thin layers were grown for calibration. In the second, five layers of InAs QDs were covered by AlAs CLs with different thicknesses.

*Materials growth*: One sample was grown by solid source molecular beam epitaxy on Si-doped (100) n+ GaAs substrate under As4-stabilized conditions. In the bottom block, 4 thin layers of AlAs were grown at 580 °C for calibration with 1, 2, 3, and 5 monolayers (MLs), respectively and separated by 30 ML of GaAs. In the upper block, over a 100-nm-thick GaAs buffer deposited at 580 °C, 5 QD layers with 2.8 MLs of InAs were deposited at 460 °C and 0.045 ML/s. Each QD layer was covered with 0, 1, 2, 3, or 5 ML of AlAs, respectively, plus 30 ML of GaAs, all grown at 480 °C. Each one is named in the following as CL0, CL1, CL2, CL3, and CL5. These capped QD layers are separated by 50 nm GaAs grown at 580 °C to avoid strain coupling. The growth rate of the AlAs and GaAs in the CLs was 0.5 and 1 ML/s, respectively. The active region was finally capped with a 200 nm GaAs layer. A scheme of the sample is showed in Figure 1. 

*Characterization methods*: The structural and compositional analysis was performed by transmission electron microscopy techniques on conventionally prepared cross-sectional samples. Diffraction contrast (DC) TEM imaging was carried out in a JEM-2100 LaB6 (JEOL, Tokyo, Japan) operating at 200 kV. Energy dispersive X-ray spectroscopy (EDX, (FEI Europe B. V., Eindhoven, The Netherlands)) was simultaneously performed in scanning (S)TEM mode with a double aberration corrected FEI Titan Cubed Themis operated at 200 kV. EDX mapping was carried out with four embedded Bruker bd-4 sx detectors (Bruker, Billerica, Massachusetts) using ChemiSTEM technology. EDX maps were processed using Velox^®^ software (Version: 2.12.1.37).

## 3. Results

### 3.1. DCTEM Analyses Using g002 DF Conditions of the CL/WL Regions

As was mentioned in the introduction, it has been suggested that the InAs WL can be reduced or even removed if a thin AlAs layer is deposited on the InAs QD layer. The experimental evidence for this claim is based on the contrast change in DCTEM imaging under g002 DF conditions. For a long time, g002 DF imaging has been widely used to visualize composition changes in III-V semiconductors [36,37]. Roughly, the diffracted intensity leaving the specimen depends on the atomic scattering factors, f, as $\;A{\left(\bar{f_{III}}-\bar{f_{V}}\right)}^{2}$, where A is essentially constant for similar alloys and TEM conditions (volume of the cell, Bragg angle, electron wavelength, sample thickness, etc.) [38,39,40]. In the case of ternary alloys, normalizing the intensities with respect to a material with a fixed composition (i.e., GaAs of the substrate) could eliminate the term A, so that parabolic-like curves of normalized intensities could be obtained as a function of the alloy composition. Thus, pure In_x_Ga_1−x_As regions appear darker if x < 0.4 as compared to the GaAs reference while the AlGaAs ones appear brighter [39].

**Figure 2:**
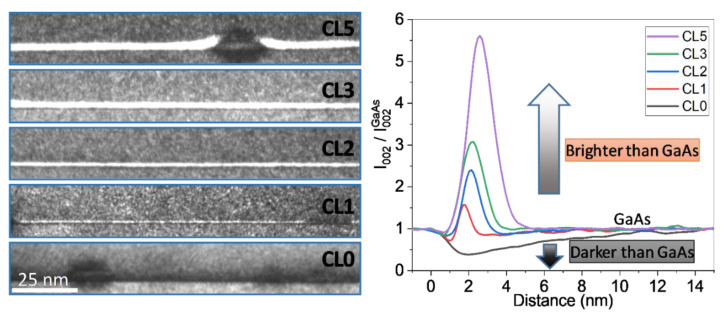
(**Left**) DCTEM g002 DF images of the QD layers with 0, 1, 2, 3, and 5 ML of AlAs CL taken close to the edge of the TEM sample to be near the kinematical conditions (sample thickness below 50 nm). (**Right**) Average intensity profiles along the growth direction of the WL regions normalized to the GaAs intensity.

Figure 2(left) shows g002 DF images of WL regions with CLs with 0, 1, 2, 3, and 5 ML of AlAs taken close to the edge of the TEM sample to be near the kinematical conditions (sample thickness below 50 nm). The dark streak of layer CL0 that corresponds to the In(Ga)As WL seems to disappear progressively being substituted by the white streak that increases as the CL thickness rises. The initial dark streak is barely observable for the layer CL5. In addition, QDs become larger in size as the CL thickness increases (see ref [41]) which is in accordance with the appearance of the shield effect that blocks the QD decomposition [30,42]. The intensity profiles normalized to GaAs for the same layers are shown in Figure 2(right). Since the profiles were normalized with respect to the GaAs substrate, Al presence gives rise to intensity ratios greater than 1 (peaks of brighter contrast) while In yields to intensity ratios were less than 1 (valleys of darker contrast) for In contents below 40% [36,39]. Valleys are only observed for the first two QD layers, but with a huge shortening for CL1. From CL2 onwards, the peak enlarges with the AlAs CL thickness. Certainly, having only these results and thinking on the nominal design of two consecutive thin layers, it seems that In atoms of the WL are being progressively substituted by Al atoms from the CL. However, as we will see below, care must be taken with this hypothesis based on the nominal design of sequential ternary layers without intermixing, since in the case of a quaternary InAlGaAs alloy, the normalized intensity regarding the GaAs depends on the relative percentage of In and Al contents and can vary from bright to dark.

Small lateral differences are observed for the same WL depending on which part is examined, and this is especially true for the more buried layers. During the thinning of the sample for electron transparency, a small thickness gradient can occur, so that the thickness of the sample in the more buried layers is thicker than that of the upper layers. Thus, the upper layers (TEM sample thickness < 80 nm) appear more homogeneous than the lower layers (TEM sample thickness greater than 120 nm) due to these thickness differences. As the distribution of QDs is random, the probability of finding a WL region between QDs where we are sure that there is no interference from neighboring QDs increases as the TEM sample becomes thinner. We had chosen this region of the sample with the intention of showing an image with all the layers. As a result, the TEM sample thickness varies along the growth direction which has the above-mentioned noticeable effect on the image. Taking all into account, DCTEM images for each layer were taken individually in regions with thicknesses below 50 nm that are near the kinematical conditions of electron diffraction.

### 3.2. EDX Analyses of the CL/WL Regions

**Figure 3:**
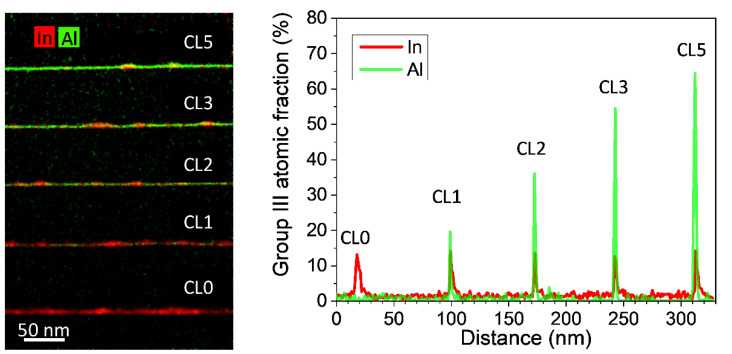
(**left**) Low magnification elemental EDX map for In and Al elements together with (**right**) In and Al atomic fraction profiles along the growth direction.

EDX analyses could supply directly interpretable elemental mappings for Al and In distribution in the structure. Figure 3(left) shows a low magnification elemental EDX map of the five QD layers where green and red hue intensities correspond to Al and In contents, respectively. As shown earlier in the g002 DF images, the nominal InAs WL regions between QD seem to fade as the thickness of the AlAs CL increases, going from a deep red hue in the CL0 layer to a deep green hue on the CL5 top layer. However, multi-channel images can be misinterpreted if both elements are in the same column position. To separate both signals, average compositional profiles along the growth direction for both elements are plotted in Figure 3(right). On the one hand, Al peaks linearly increase with AlAs thickness and considering the typical segregation in these systems, the AlAs is incorporated in the regions between QDs in a quantity proportional to the intended AlAs deposition. On the other hand, In peaks are always present and show similar maximum contents (around 13–15%) for all five layers although they become narrower as AlAs deposition increases. These results point out that the total amount of InAs in the WL regions may decrease in the different QD layers but not until its complete deletion. 

**Figure 4:**
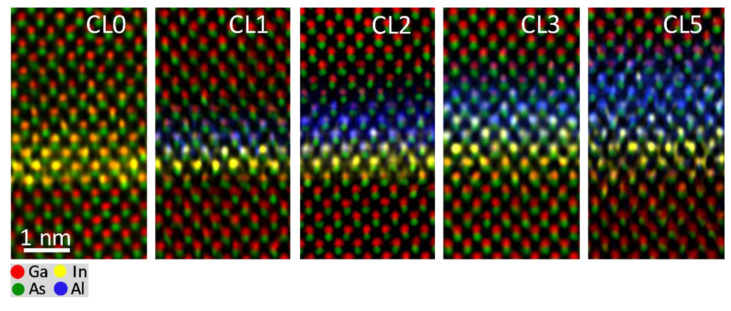
Atomic-level-resolution EDX elemental maps of WL/CL regions for the different InAs QD layers with 0, 1, 2, 3, and 5 ML of AlAs acquired along the [110] zone axis.

EDX mappings at low magnification have proved that the WL is not eliminated, but rather a strong superposition between In and Al profiles is present. However, at this magnification, small shifts during EDX map acquisition could lead to mistaken conclusions with respect to spatial distribution and the maximum content detected. With the aim of investigating the overlapping between WL and CL regions, EDX analyses with atomic-column-resolution of the CL/WL region were carried out for the different QD layers using drift correction software during acquisition (Figure 4), where red, yellow, green, and blue colors correspond to Ga, In, As, and Al atoms, respectively. Channeling, probe scattering, and fluorescence could influence the atomic detection and quantification in high spatial resolution EDX maps. A compromise between all parameters (thickness sample, converge angle, etc.) is necessary to ensure practical confidence in the results [43]. At the expense of a certain hindrance in quantitative interpretation, a small convergence angle (16 mrad) was used in areas of the TEM sample with thicknesses between 20 and 40 nm. These column resolved EDX elemental maps allow resolving the dumbbell structure in which As anions are at the lower position and cations (Ga, In, or Al) in the upper. Ga atoms are predominant in the areas at the top and bottom of the image while the In and Al atoms are in the center. Note that In atoms presence seems to appear a little bit before blue fringes linked to Al, which expand as the AlAs thickness increases.

**Figure 5:**
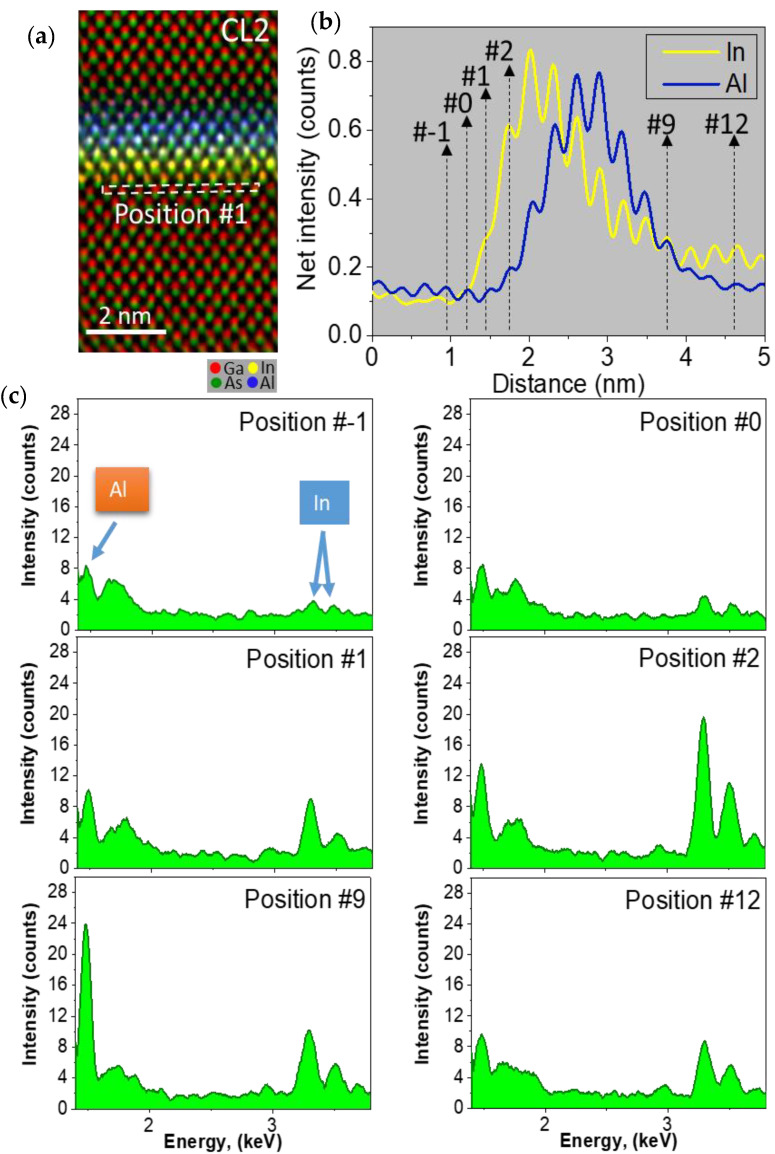
For the WL of CL2, (**a**) EDX map showing the distribution of Ga, In, As, and Al. A rectangular ROI is showed at position #1 (**b**) Net counts profiles for In and Al along the growth direction. (**a**,**c**) EDX spectra for ROI displacements along the growth direction in position #−1, #0, #1, #2, #11, and #12. Spectra of 4096 points in length were smoothed on the graph using Savitzky-Golay algorithm using a 30-point window to remove some background noise.

To know the exact positions where In (of the WL) and Al atoms (of the CL) begin to be incorporated, a careful layer by layer study of the EDX spectra has been performed for each QD layer. Thus, for the case of the QD layer CL2, Figure 5 shows (a) an EDX map and (b) the average elemental profiles along the growth direction for In and Al using net counts. In these profiles, each peak corresponds to the position of every atomic layer in the growth direction. Considering this, we defined a rectangular region of interest (ROI) with the height of 1 cation width in (a) as reference position 1. Figure 5c shows different EDX spectra obtained by shifting this ROI to distinct positions along the growth direction. First, the position number #-1 corresponds to the GaAs buffer without In and Al, just before the beginning of the InAs WL. Spectra taken on inferior positions (not shown here) present a high similarity, which discard the possibility of a significant channeling effect of the upper layers with Al and In on the spectrum of position number #-1. Since the presence of spurious peaks at 1.487 keV (Kα1 for Al) and 3.279 keV (Lα2 for In) in the GaAs spacer also appear on the GaAs substrate spectra, they are considered as artefacts (fluorescence and/or backscattered electrons from support grid, objective pole-pieces, surface contamination, etc.,) which do not correspond to the X-rays generated by the specimen [44]. Thus, the signal of In starts in position number #1, where there is no evidence of Al atoms. From position #2 onwards, the Al signal appears coexisting with the In one. It should be noted that there is a delay of only 1 ML in the beginning of the incorporation of Al with respect to In. Al atoms hardly reach position #12 but In atoms do, being present until position #15.

**Figure 6:**
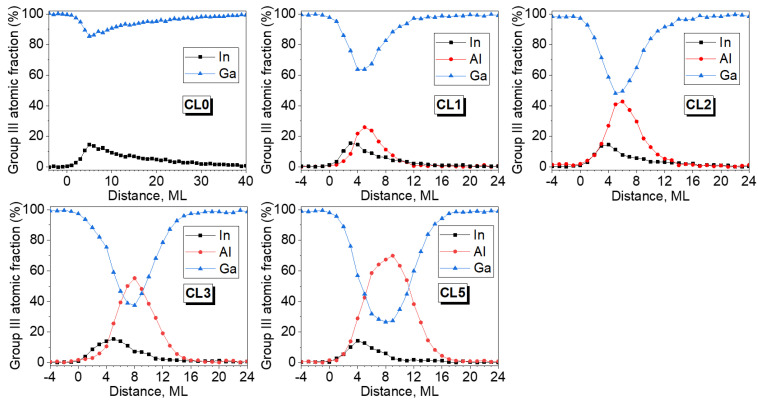
Compositional profiles at ML resolution level for elements of the III group along the growth direction for the WL region in the different AlAs/InAs QD layers.

Several composition profiles, from regions with sample thicknesses below 50 nm, along the growth direction were averaged using different EDX maps for each QD layer. The counts were discretized considering the sequential monolayer structure and the In, Ga, and Al contents were calculated for each ML. The compositional profiles along the growth direction in different regions are remarkably similar, so we deduce that these column resolved EDX maps are representative of the overall behavior for each QD layer. Figure 6 shows the results of these profiles in WL regions without QDs for each QD layers. Focusing on the In profiles, all In curves show the effects of surface segregation with a typical shark-fin shape. For all layers, the In-content increases during the first 3–4 MLs reaching up to 15–17% and then slowly diminishes in an exponential way. All the In profiles are remarkably similar at the beginning, almost independent of the amount of the AlAs deposited, the differences are found during the decline after the maximum. The number of MLs with the presence of In is reduced almost four times, falling from more than 40 ML for the case without AlAs CL (CL0) down to ~10 MLs for the QD layer CL5. According to these results, there is a reduction of the In amount in WL but only in the decay part. 

On the other side, Al profiles are almost symmetrical displacing the peak maxima to taller positions when Al deposition is higher, from the position #5 up to position #10 for CL1 and CL5 layers, respectively. Comparing both In and Al profiles, it can be seen in every case that the Al peak maximum is displaced with respect to the In peak a distance proportional to the CL thickness. The Al contents are always higher than the In ones when the In decay begins and even in the growing part of the profiles from 2 ML of AlAs onwards. The great overlapping between both profiles together with the reduction of the In tail would explain the contrast changes in the g002 DF images as the AlAs CL thickness increases. Of note is the significant continuous presence of Ga atoms throughout the CL/WL region, always exceeding 30% of the III-sites. The regions between QDs must be considered as a quaternary InAlGaAs alloy with a variable composition where In is the minority component. 

### 3.3. Simulation of g002 DF Images

**Figure 7:**
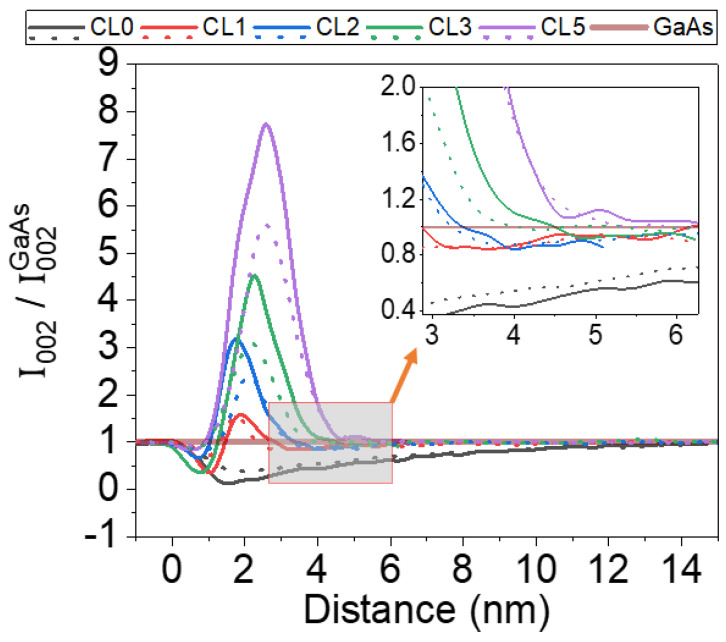
Normalized profiles of the intensities in g002 DF images of WL/CL region along the growth direction simulated using the compositional profiles of Figure 6 (solid line) and experimental g002 DF images (dot line) for the different AlAs/InAs/GaAs QD layers. The inset shows the presence of a second darker streak after the brighter AlAs contrast due to In surface segregation.

As we have commented above, the intensities of g002 DF images for semiconductors with zinc blende structure are strongly sensitive to local composition changes and are poorly coupled to the local strain [45]. These images are quite simple to simulate using only atomic scattering factors in kinematical conditions (sample thicknesses below ~50 nm), albeit at semi-quantitative level [38]. Figure 7 shows the simulation of the corresponding diffracted intensity under two-beam kinematic g002 DF conditions normalized to GaAs of WL/CL region using the compositional profiles from Figure 6 together with the experimental profiles extracted from g002 DF images (Figure 2(Left)).

For the CL0 layer, the WL in g002 DF image conditions appear as a wide dark band expanding up to 12 nm with intensities less than 1. The addition of only 1 ML of AlAs changes the profile intensely. The thickness of the contrast of the WL/CL is reduced to 3 nm with a first thin dark valley followed by a thin bright peak. With the AlAs incorporation, the first dark valley progressively vanishes, increasing the area of the peak associated with the bright contrast. Simulations even predict the presence of a second dark stripe after the white band (see the inset of Figure 7). This second stripe occurs because the segregation of the In atoms surpasses the position of the AlAs layer. The simulation is in remarkably good agreement with the experimental profiles of Figure 2. Certainly, experimental measurements of the g002 DF intensity even for ternary layers do not exactly match the simulations, probably because it is sensitive to other parameters such as the deviation parameter from the Bragg condition, contributions from 004 reflection, amorphous layers on the sample surface, strain fields, and so on. All attempts to advance the quantification of these materials using DCTEM images have never provided reliable results [39]. In any case, the high intermixing between the CL and the WL from the very beginning of deposition and the increasing amount of AlAs with respect to InAs explain the “apparent” dissolution of the WL. 

### 3.4. Global Elemental Quantification of the WL and CL

**Figure 8:**
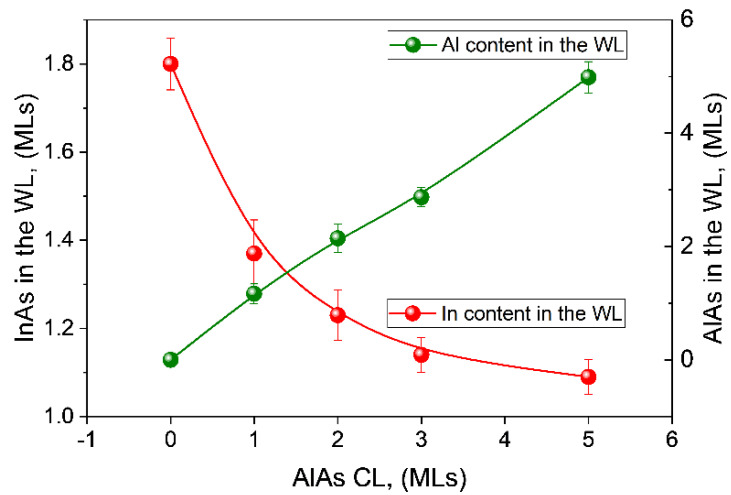
In and Al amount in the CL/WL regions expressed as deposited MLs. The results are obtained from the calibrations of the area in averaged profiles along the growth direction from EDX measurements in thin layers. Error bars shows the upper and lower 95% confidence limits using the standard error for the sample mean, multiplied by 1.96.

In a thin layer, the area under the EDX compositional profiles along the growth direction has been confirmed as the best parameter for assessing the total amount of material deposited. Previous calibration with different InAs thin layers without QDs permitted us to quantify the total amount of In that remained in the WL regions of QDs layers expressed in MLs of pure InAs [46]. For the case of AlAs layers, similar method was applied in this work, using the first layer block of the sample for calibration. Figure 8 displays the global In and Al amounts of the CL/WL expressed as deposited MLs using this procedure for every QD layer. On the one hand, In amounts in the WL show an exponential decay with an important reduction in comparison with the InAs deposited in CL0 (1.8 ML). The global In content seems to reach a plateau in layer CL5, so we should not expect further reductions for InAs amounts in the WL with increasing thickness of the AlAs CL. This decrease in In content in the WL implies that a higher percentage of the In content is kept in the QDs. This result agrees with previous data [41], which showed a progressive increase in the heights and average content of the QDs as the thickness of the CL increases. It is proposed that AlAs capping has a large shielding effect against the decomposition of QDs, since it presents a significant percentage of pyramidal QDs, which is their stable form before capping. Moreover, in the case of very thick CLs (>5 MLs), the excessive shielding of AlAs against the erosion of QDs has detrimental effects because it preserves the giant QDs existing in uncapped surfaces.

Contrary to the large variations of In content in the WL regions between QDs during the capping process, the amount of Al in these regions follows a linear trend that agrees with the nominal number of MLs introduced during growth. That means that Al is homogeneously spread on the surface without significant accumulation in the QDs and regardless of the changes that occur in the QDs. This is consistent with the higher energy barrier for surface diffusion of Al (0.8 eV) [47] compared to Ga (0.62 eV) [48] or In (0.4 eV) [49]. At these growth temperatures, surface diffusion lengths ($L=\sqrt{D_{s}\tau }$, where Ds is the surface diffusion coefficient and τ the transient time), are of the order of a few, dozens, or hundreds of nm for Al, Ga, and In, respectively. In atoms are very mobile, while Al atoms are almost immobile, staying close to the first surface position where adsorption occurs. Since Al atoms show the shortest surface displacements once adsorbed on the surface at this temperature, the degree of coating of QDs from the first stages of CL growth is remarkably high. The nearly immobile Al atoms rapidly cover the surface of the QDs, freezing the surface redistribution of In and slowing down the loss of In by desorption from the QDs during overgrowth. Consequently, the mass transfer of In to the WL from the QDs is reduced, resulting in larger QDs and thinner WLs [41].

## 4. Discussion

### 4.1. WL Reduction

The deposition of a thin layer of AlAs on InAs QDs layers has been found to work as a way to improve the *V*_OC_ in InAs/GaAs QDSCs [14,18], where it has been suggested that the elimination of the WL during the capping process of AlAs is its cause [22]. Our work has shown that a very thin layer of AlAs induces a significant reduction of the WL thickness but not its complete removal. Thus, the WL of the CL5 layer only keeps an amount of In equivalent to 1.1 MLs of InAs compared to the initial 2.8 MLs deposited. Remarkably, this reduction of the WL is stronger than in other capping strategies such as the use of strain-reducing layers (SRL) or increased GaAs CL growth rate [34,50]. On the one hand, the amount of InAs in the WL after accelerating the growth rate of the GaAs CL to 2 ML/s is 1.7 MLs of InAs, but no substantial improvement is expected for faster growth [46,51]. On the other hand, using a SRL of GaAs_0.8_Sb_0.2_, which is almost the high Sb content limit for GaAsSb capping without plastic relaxation, results in a WL with 1.4 ML of InAs [34]. Certainly, capping with AlAs entails a further reduction of the WL but in line with what has been observed with other materials.

In addition, the analysis of the profiles allows us to assess the Tsatsul’nikov’s hypothesis of migration of In toward the dots by substitution of In for Al in the WL [22]. As can be seen by comparing the In profiles in Figure 6, the upward sections of the curves are almost identical for all layers, reaching the same maximum, while the downward sections differ, corresponding to the segregation tail of the In accumulated in the floating layer. Therefore, only this decay section takes part in the comparison of the WL mass reduction. First, the fact that the ascending sections of the In profiles are the same is not compatible with a mechanism of substitution of In for Al in the WL. Al is present from the beginning and in different amounts, so the incorporation of Al in the CL does not affect the already incorporated In profile. Second, the decrease of the downward profile cannot be explained by the supposed migration of In from the WL to the QDs either. All segregation models to describe the decay profiles due to surface segregation in InGaAs QWs [25,27] cannot be applied for the case of WLs in InAs QDs systems. The reason is that the segregation profiles in the case of QWs are always much smaller, being necessary to add an extra amount of In to correctly fit the segregation tails of the WL profiles in QD systems [35]. This added amount of In can only come from the decomposition of the QDs, where it has been found that there is a relationship between the size of the QDs and the In content in the WL. The larger the average QD size, the thinner the WL between the QDs [34,46]. Therefore, the longer segregation tail of the WL without AlAs capping (CL0) is explained by the accumulation of In from the greater decomposition of its QDs, which showed the smallest size. In contrast, shorter tails of In profiles in the case of thicker AlAs layers are linked to the presence of larger QDs, with shapes and sizes similar to uncapped surface QDs [41].

As we have seen, capping with AlAs occurs with processes like those seen with other alloys. First, individually, QDs undergo a decomposition, changing their structure by shortening their heights and reducing their In content and deformation state [52,53,54]. One of the main driving forces is the intermixing process due to the lattice mismatch between the QDs and the WL, leading to mass transport away from the QDs during capping [55]. Second, collectively, there could be a significant decrease in the QD density after capping [34,56]. Therefore, the region with the WL away from the QDs becomes enriched with In due to lateral migration from the decomposition of the small QDs [35,50,57]. Our results suggest that capping with AlAs does not remove In from the WL during the capping as it has been proposed but acts against the QD decomposition hindering the surface diffusion of In from the QDs and reducing the amount of In available for surface segregation in the WL region.

### 4.2. CL/WL Mixing

Though the WL reduction is considerable, it is not the only reason for the V_OC_ recovery. A key factor could be the high intermixing showed in our work that happens between the AlAs CL and the InAs WL. EDX profiles at atomic resolution of the In and Al content along the growth direction showed a high degree of overlapping from the first MLs, that is, there is almost no physical separation between these layers with a defined interface but only one graded layer with variable content of these elements. The first 3–4 MLs of this InAlGaAs layer exhibit almost superposed profiles with similar contents of In and Al, but in the next layers, the peak of the Al profile always surpasses the peak of In, even for the sample with 1 ML of AlAs (see Figure 6). 

This result is consistent with the small thickness of the WL before capping. The deposition of 2.8 ML of InAs is in fact distributed between the QDs and the WL regions. As can be seen in Figure 8, the amount of InAs in the WL in the case of the layer CL5 is only a little higher than 1 ML. In the case of the layer CL0, the In amount is higher (about 1.8 ML) but this is due to the added incorporation of In due to the QD decomposition process during capping that yields a long tail in the profile. Our results point to a thickness of InAs in the WL before capping of approximately 1 ML or less. During AlAs capping, Al incorporation occurs over this WL of 1 ML thickness. From here, Al and In from the floating segregated layer are incorporated simultaneously. This explains the huge overlap of both profiles with only a small lag of 1 ML (see Figure 6) at the onset of InAs growth without the need for any substitutional mechanism.

In addition, the high simultaneity of both profiles could be explained by the surface segregation energies. Surface atomic exchanges require overcoming energetic barriers for bulk-to-surface switches that depend on the cohesive energies in the case of III-V compounds [24,58], being 178.9, 154.7, and 144.3 kcal/mol for AlAs, GaAs and InAs, respectively [59]. Then, Al/Ga exchange favors surface segregation of Ga instead of Al [60], while In is always strongly segregated to the surface in the presence of both Ga and Al [61]. As a result, Al atoms, which are incorporated on the first ML of the WL, are in comparison sessile while In always segregates, crossing the AlAs layer and overcoming it.

However, the situation is somewhat different for other CLs of a different nature, such as GaAsSb, where there is a large shift of the CL profile with respect to the WL profile [62,63]. The reasons are multiple. First, the lower cohesive energy of GaSb (138.6 kcal/mol) compared to InAs [59], which means a higher surface segregation of Sb with respect to In. Second, the lower nominal contents of GaAs_1−*x*_Sb*_x_* CLs (*x* < 0.2) implies that the amount of Sb incorporated under the surface at the beginning of the CL growth is low, since the surface can accommodate significantly more Sb before saturating [27]. Consequently, the two profiles have little overlap, and there is even an intermediate zone rich in Ga between both.

### 4.3. Bandgap Approximation of the CL/WL Regions

Finally, the high intermixing and the reduction of the In content in the regions between QDs have a noticeable impact on the electronic states. Due to the difficulty in preparing perfectly controlled InAlGaAs QW structures, little experimental data are available to obtain a set of material parameters for estimating the bandgap in the InAlGaAs system. Because of this limitation, only two equations for the room temperature dependence of the bandgap have been obtained experimentally [64,65], which have a realistic agreement with the published expression for AlGaAs, InGaAs, and AlInAs ternary alloys. Due to the lack of experimental studies at low temperatures, we have used the low-temperature bandgap relation for the InAlGaAs system proposed by E. H. Li [66], based on an interpolation method using the material parameters of the ternaries [67]. In general, a good agreement between the calculated and experimental results over a wide range of QW width and alloy concentration is achieved in the QW structures of III-V alloys using these approaches.

**Figure 9:**
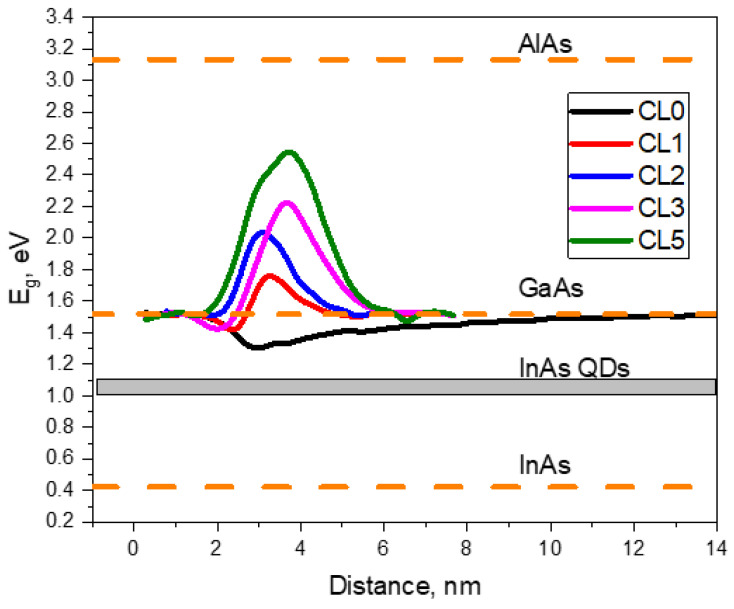
Bandgap approximation at 4.2 K of the WL/CL system along the growth direction, for the different layers in the regions between QDs.

Figure 9 shows the evolution of the bandgap energy (*E*_g_) at 4.2 K along the growth direction for the different layers in the regions between QDs using the EDX compositional profiles and considering the interpolated bowing factor for an InAlGaAs alloy [66]. For the CL0 layer, the WL works as a quantum well with an energy minimum around 1.3 eV.

For comparison, the experimental bandgap of regular InAs QDs ranges from 1 to 1.15 eV [14,16,19]. The energy gap between the WL and the QDs protects the electrons and holes of QDs from coupling, but only to a certain extent, since a low-energy tail of the WL continuum can extend to the confined states of QDs. This energy gap can be bridged by many phenomena [16], which has negative consequences for quantum applications. However, the deposition of very thin layers of AlAs changes the behavior radically. With the introduction of AlAs, the energy well of the WL/CL shrinks largely until it disappears and a potential barrier for electron and holes is additionally formed. As can be appreciated in Figure 9, the formation of an InAlGaAs layer shifts the bandgap to higher energies, limiting the undesirable hybridization of states between the WL and the QDs. This barrier grows with increasing CL thickness, which could be detrimental for carrier transport in the solar cell [16] but is far from the values expected for pure AlAs layer (3.13 eV). In this sense, the large intermixing reduces not only the depth of the potential well due to In, but also limits the height of the barrier due to Al. Remarkably, the layers with 1–2 mL of AlAs show the most compensated configurations, avoiding, on the one hand, big barriers achieved with large CL thicknesses and, on the other hand, deep potential wells which can be observed without AlAs.

## 5. Conclusions

The use of thin layers of AlAs in InAs QDSC devices has been proposed to improve the open circuit voltage by intending to make the WL vanish. In this work, the structure of the CL/WL regions between QDs has been characterized at the atomic column level applying the CL thickness variation. First, our results have shown a significant reduction of the WL due to a QDs decomposition reduction, but far from its complete elimination such as those suggested in earlier TEM works. Second, both CL and WL show a high intermixing from the first layers resulting in an InAlGaAs layer with gradual composition. The latter explains the mistaken dissolution of the WL proposed in the CDTEM studies using g002 DF conditions. Both factors cause a drastic change in electronic levels, from a QW for the case without AlAs to a huge barrier for the case of thickest CL, the CL with 1–2 ML being the most adequate configuration for SC applications.

## Data Availability

Not applicable.
